# Subgingival periodontal pathogens associated with chronic periodontitis in Yemenis

**DOI:** 10.1186/1472-6831-14-13

**Published:** 2014-02-18

**Authors:** Nezar N Al-hebshi, Hussein M Shuga-Aldin, Ali K Al-Sharabi, Ibrahim Ghandour

**Affiliations:** 1 Department of Preventive Dentistry, Faculty of Dentistry, Jazan University, P.O. box: 114, Jazan, Saudi Arabia; 2 Molecular Research Laboratory, Faculty of Medical Sciences, University of Science and Technology, Sana’a, Yemen; 3 Department of Periodontology, Oral Pathology, Oral Medicine and Radiology; Faculty of Dentistry, University of Sana’a, Sana’a, Yemen; 4 Department of Periodontology, Faculty of Dentistry, Khartoum University, Khartoum, Sudan

**Keywords:** Microbiology, Pathogens, Real-time PCR, Parvimonas micra, Periodontitis

## Abstract

**Background:**

Subgingival microbial profile associated with periodontitis has been reported to significantly differ by geographical location. The purpose of this study was to assess the association between a panel of putative periodontal bacterial pathogens and chronic periodontitis among Yemenis.

**Methods:**

Subgingival DNA samples were obtained from diseased and healthy sites of 20 non-smoking, moderate to severe chronic periodontitis subjects. Absolute counts (bacterial DNA copies per sample) and relative counts (% total bacteria) of seven periopathogenic species/genera representative of the red and orange complexes were determined using Taqman q-PCR assays.

**Results:**

The q-PCR assays showed excellent linearity (R^2^ > 0.99) and a sensitivity of 100 copies/sample. The detection rate was 100% for all tested species/genera except for *P. gingivalis* and *A. actinomycetemcomitans* that were detected at 97.5% and 67.5%, respectively. The median log absolute counts were in the range of 2.41-6.53 copies per sample while median relative counts were in the range of 0.001-0.77%, both being highest for fusobacteria and lowest for *A. actinomycetemcomitans*. Significant interspecies correlations were observed. Adjusting for multiple comparisons (P≤0.0063), only *T. forsythia, T. denticola* and *P. micra* maintained significant association with periodontal destruction. The latter species, however, showed the strongest association and was found in higher proportions at the periodontitis sites across all subjects (3.39 median fold increase). No significant differences were observed for *P. gingivalis.*

**Conclusions:**

*P. micra* rather than *P. gingivalis* appears as a keystone pathogen in this Yemeni Sample. However, these findings need to be validated in a larger-scale study before they can be claimed to represent ethnic variations in pathogens’ association with periodontitis.

## Background

Periodontitis represents a range of clinical entities that are characterized by immunological destruction of the tooth supporting structures in response to chronic challenge by specific bacteria in subgingival biofilm [1]. The last three decades or so witnessed an explosion in our understanding of the microbiology of periodontitis. Earlier studies employing cultivation-based techniques recovered about 250 bacterial species (at > 1% relative abundance) from plaque samples [2]. With the extensive application of molecular techniques over the last decade, double this number of novel species has been identified [3-5] bringing the richness of the subgingival microbiota to more than 700 bacterial species, about 50% of which are uncultivable.

While the majority of subgingival microbiota is considered commensal, several species have been implicated as periodontal pathogens. *Porphyromonas gingivalis*, *Tannerella forsythia*, and *Treponema denticola* (the so called red complex) have so far shown the strongest association with chronic periodontitis [6]. Other putative pathogens include *Fusobacterium* spp., *Prevotella* spp., *Campylobacter rectus*, *Eubacterium nodatom*, and *Parvimonas micra* (previously *Peptostreptococcus micros*) [6]. Uncultivable phylotypes such as *Synergistetes*, TM7 and *Treponema* taxa are also believed to play a pathogenic role in chronic periodontitis [3,4,7]. In fact, it is believed that periodontal destruction is triggered by a bacterial consortium rather than a single pathogen [1].

A number of molecular techniques have been employed for detection and quantification of periodontal pathogens in plaque samples including DNA-DNA hybridization, conventional and real time PCR, and 16S rRNA clone sequencing [8]. Of these, real-time PCR is the most sensitive allowing detection of as low as 1.6 cells per reaction [9,10]. It also makes it possible to normalize target DNA counts to total bacterial counts in the sample (relative quantification), thus adjusting for variations in sampling and making comparisons between samples more reliable [11,12]. Surprisingly, real-time PCR has not been as widely used in the study of microbiology of periodontitis as may be expected.

Subgingival microbial profile associated with periodontitis have been reported to significantly differ by geographical location independent of other factors known to modify subgingival microbial composition [8,13]. It becomes prudent, therefore, that obtaining more information about the global distribution of periodontal pathogens and patterns of their association with disease can improve our understanding of the differences in the role they play in periodontitis in different populations. In the absence of data on this from the Middle East, the objective of the current study was to assess the association of seven putative periodontal pathogens with chronic periodontitis in a Yemeni population using quantitative PCR assays.

## Methods

### Study subjects and clinical examination

Twenty subjects, 30–50 years old, with moderate to severe chronic periodontitis (having at least 1 site per quadrant with pocket depth ≥ 5 mm and attachment loss > 3 mm), were recruited from among patients attending dental clinics at Al-thawra hospital, Sana’a, Yemen. Subjects presenting with less than 20 teeth or diagnosed with aggressive periodontitis (those with typical first molar/central incisor presentation) were excluded. Other exclusion criteria included history of smoking, periodontal treatment or antibiotic/oral antiseptic use in the previous 6 months, pregnancy or breast feeding, and any systemic disease or medication intake known to modify periodontal inflammation.

The community periodontal index [14] was used to screen periodontal status by a single, well-trained and precaliberated examiner (Shuga-aldin HM). In eligible subjects, pocket depth (PD) for the deepest pocket in each quadrant in millimeters was established using a Williams probe. The plaque index [15], was measured on the labial/buccal and lingual/palatal surfaces of index teeth. The clinical characteristics of the study group are shown in Table 1.

**Table 1:** Clinical characteristics of the study group

Gender (M/F %)	60/40
Age, median (interquartile range)	40 (30–45)
Plaque index, median (interquartile range)	1.5 (1.25-1.65)
Pocket depth at sampled sites, median (interquartile range)	5.5 (5.00-6.75)

The study was carried in compliance with the Helsinki declaration. It was approved by the Medical and Health Studies Board, Graduate College, Khartoum University. Informed consent was obtained from all subjects.

### Sampling and DNA extraction

For each subject, one pooled subgingival sample from the deepest pocket in each quadrant (PD ≥ 5 mm) and another from 4 healthy sites (PD ≤ 3 mm; no attachment loss) were obtained, using sterile paper points. Supragingival plaque was removed prior to sampling using sterile cotton pellets. The samples (40 in total) were stored in low EDTA TE buffer (Invitrogen, USA) at -80°C until processing.

At the time of DNA extraction, samples were centrifuged at 15,000 g for 1 minute and the pellet was resuspended in 180 μl lysozyme digestion buffer (25 mM Tris–HCl, pH 8.0, 2.5 mM EDTA, 1% Triton X-100) containing 20 mg/ml lysozyme, and incubated at 37°C overnight. The digest was then subject to DNA extraction using the Purelink Genomic DNA extraction kit (Invitrogen, USA); DNA was eluted in 100 μl of the supplied buffer and stored at 4°C for subsequent analysis.

### Quantitative PCR assays

Total bacteria, *Fusobacterium* spp., *Prevotella* spp., *Aggregatibacter actinomycetemcomitans* (previously *Actinobacillus actinomycetemcomitans*), *Parvimonas micra* (previously *Micromonas micra* or *Peptostreptococcus micros)*, *Porphyromonas gingivalis*, *Tannerella forsythia*, and *Treponema denticola* were detected and quantified in the DNA extracts using Taqman real-time PCR technology [16]. Sequences of probes and primers used in the study are shown in Table 2. They were supplied by Primerdesign, UK, as optimized and ready to use kits which also included plasmid-based positive control (amplicon sequence inserted). The latter was serially diluted to construct standard curves for absolute quantification of the test species, and to assess efficiency, linearity and sensitivity of the assays.

**Table 2:** Sequences of primers and probes used in the quantitative PCR assays

**Test species**	**Sequences 5′-3′**	**Target gene**	***Product size***	***Ref***
*A. actinomycetemcomitans*	F-primer: GGRAGAATGGATGGCGATAT	hgpA	81 bp	This study
	R-primer: ATCAGAATGAACATAACCTATACCA			
	Probe: FAM- ATGAACGCAATTCAGCCCAGA ACTG-BHQ			
*P. micra*	F-primer: TGAGCAACCTACCTTACACAG	16S rRNA	112 bp	[17]
	R-primer: GCCCTTCTTACACCGATAAATC			
	Probe: FAM- ACCGCATGAGACCACAGAA TCGCA-BHQ			
*P. gingivalis*	F-primer: ACGAATCAAAGGTGGCTAAGTT	fimA	85 bp	[17]
	R-primer: TTAGTCGCATTTTCGGCTGAT			
	Probe: FAM- CCTGCTGTTCTCCATTATAAAC CATTACGG -BHQ			
*T. forsythia*	F-primer: GATAGGCTTAACACATGCAAGTC	16S rRNA	99 bp	[17]
	R-primer: GTTGCGGGCAGGTTACATAC			
	Probe: FAM- TTACTCACCCGTGCGCCGGTCG-BHQ			
*T. denticola*	F-primer: GGGCGGCTTGAAATAATRATG	16S rRNA	92 bp	[17]
	R-primer: CTCCCTTACCGTTCGACTTG			
	Probe: FAM- CAGCGTTCGTTCTGAGCCA GGATCA-BHQ			
Total bacteria	F-primer: AAACTCAAAGGAATTGACGGGG	16S rRNA	205 bp	[17]
	R-primer: TTGCGCTCGTTGCGGGACT			
	Probe: FAM-CTGTCGTCAGCTCGTGTCGTGA-BHQ			
*Fusobacterium* spp.*	F-primer: CGCAGAAGGTGAAAGTCCTGTAT	23S r RNA	101 bp	[18]
	R-primer: TGGTCCTCACTGATTCACACAGA			
	Probe: FAM- CTTTGCTCCCAAGTAACATG GAACACGA-BHQ			
*Prevotella* spp.*	F-primer: ACCAGCCAAGTAGCGTGCA	16S rRNA	153 bp	[19]
	R-primer: TGGACCTTCCGTATTACCGC			
	Probe: FAM- AATAAGGACCGGCTAATTCC GTGCCAG -BHQ			

*Species coverage is provided in the original reports.

To check for specificity, primers’ sequences were first blasted against eubacterial sequences database at the National Center for Biotechnology Information (NCBI; http://www.ncbi.nlm.nih.gov/tools/primer-blast/index.cgi?LINK_LOC=BlastHome). Then, each set was tested in a SYBR Green real-time PCR assay against a pooled subgingival DNA sample from 5 periodontitis patients, followed by disassociation curve analysis. A primer set was judged as being specific if it resulted in a single disassociation peak that is identical to the positive standard peak.

Each reaction comprised of 10 μl mastermix with ROX (Primerdesign, UK), 1 μl primers/probe mix, 5 μl template DNA (or positive standard), and 4 μl PCR-grade water; all runs were carried out on an ABI 7000 real-time PCR platform (Applied Biosystems, USA) using the following program: initial enzyme activation at 95°C for 10 min followed by 40 cycles of denaturation at 95°C for 15 seconds and annealing/extension at 60°C for 1 min. Data were acquired through the FAM channel.

Absolute counts, in copies/reaction, were calculated using the standard curves; these were then converted into copies/sample by multiplying by 20 (since 5 μl of the extract was included in the reaction). Relative counts of the test species/genera were then calculated as % total bacteria.

### Statistical analysis

Examining clinical and microbiological data with the Kolmogorov-Smirnov statistic revealed non-normal distribution. Consequently, they were summarized as medians and interquartile ranges (IQR). Significance of differences between healthy and periodontitis sites in terms of absolute (log-transformed) and relative counts were sought using the Wilcoxon-signed rank test. Bonferroni’s correction for multiple comparison was applied so an adjusted p-value of 0.0063 was used to describe significant difference. All tests were performed using SPSS 17 (SPSS Inc., Chicago, IL, USA).

## Results

### The quantitative PCR assays

**Figure 1:**
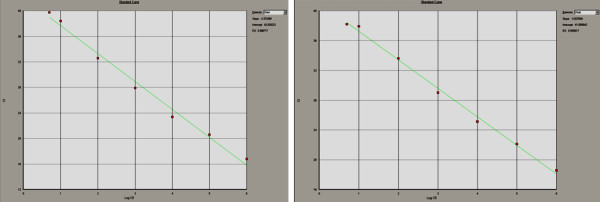
**Screen shots of ABI 7000 SDS software’s output showing standard curves for two of the primers/probe sets used in this study (*T. denticola *to the left and *A .actinomycetemcomitans *to the right).** Serial dilutions of plasmid-based positive control were prepared with final concentrations of 5-10^6^ copies/reaction. Assays were run as described in the text. The curves were obtained by plotting log DNA copies count against threshold cycle values (C_t_).

**Figure 2:**
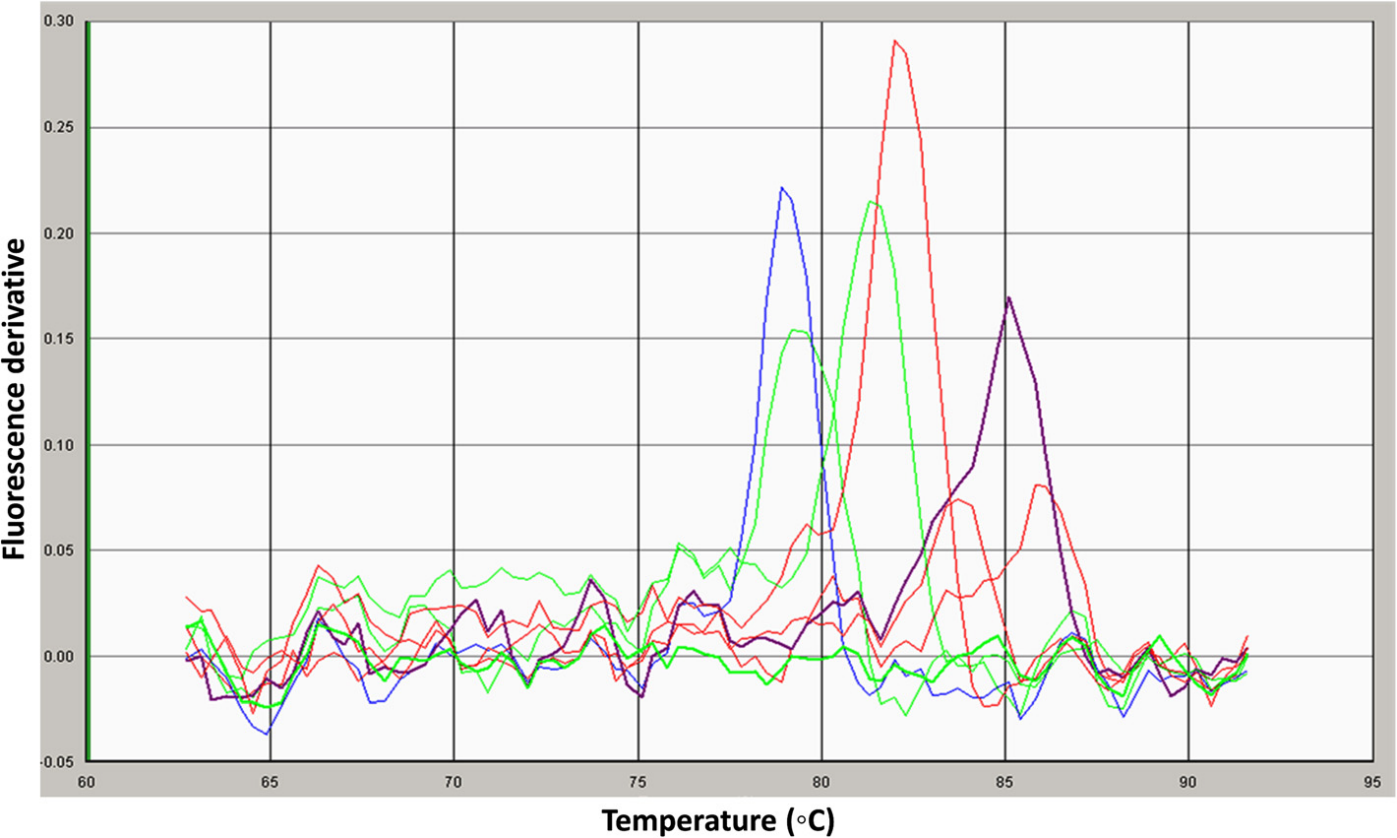
**Disassociation curve analysis of amplicons produced by testing each of the primer pairs used in the study against pooled subgingival DNA sample from five periodontitis subjects using a Syber Green PCR assay.** Each pair resulted in only one peak indicating specific amplification.

All eight real-time PCR assays showed excellent linearity (R^2^ ≥ 0.99) over a dynamic range of 5-10^6^ copies/reaction (Figure 1), achieving a sensitivity of 100 copies/sample (given DNA extraction was 100% efficient). All primer sets produced single disassociation peaks in the SYBR Green assays that corresponded to the standard peaks (Figure 2).

### General microbiological findings

**Figure 3:**
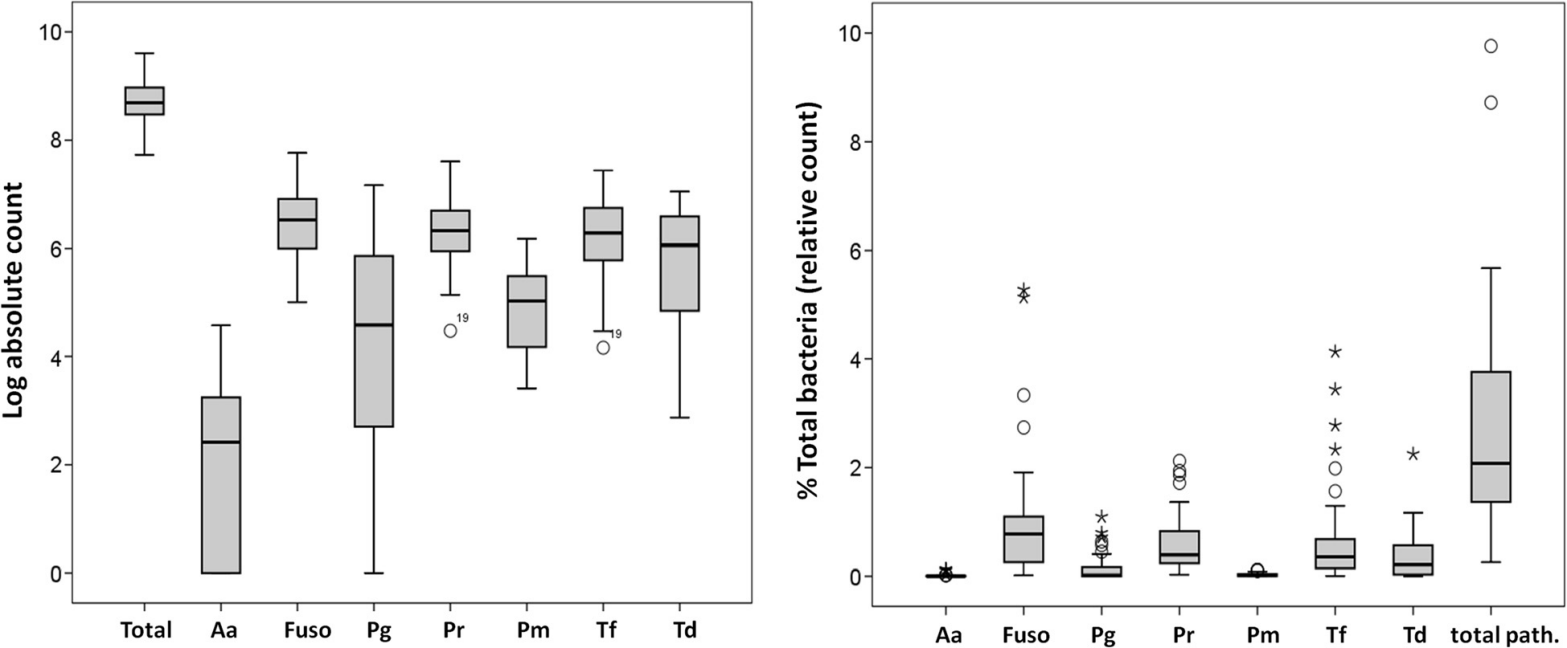
**Box plots showing the median and interquartile range (IQR) of overall absolute (left) and relative (right) counts of test species/genera in subgingival biofilm samples.** The error bars represent data within 1.5 IQR above Q3 (third quartile) and below Q1 (first quartile). Circles and stars are outliers. Aa; *A. actinomycetemcomitans*; Fuso: fusobacteria; Pg: *P. gingivalis*; Pr: prevotellae; Pm: *Parvimonas micra*; Tf: *T. forsythia*; Td: *T. denticola.*

All tested species/genera were detected in 100% of the samples except *P. gingivalis* and *A. actinomycetemcomitans*, for which the detection rates were 97.5% and 67.5%, respectively. Overall absolute and relative counts data are presented in Figure 3. The median log absolute count was 8.69 for total bacteria and in the range of 2.41-6.53 for individual species, being highest for fusobacteria and lowest for *A. actinomycetemcomitans*. Median relative counts (% total bacteria) were in the range of 0.001-0.77%, again being highest for fusobacteria and lowest for *A. actinomycetemcomitans*. Total pathogens (sum of all 7 species/genera) constituted 2.1% (IQR 1.36-3.87%). No species was detected at higher than 1% in 75% of the samples. *A. actinomycetemcomitans, P. gingivalis* and *P. micra* were never detected at more than 1% while fusobacteria, prevotellae, *T. denticola* and *T. forsythia* reached as far as 5.3%, 2.1%, 2.3% and 4.1%, respectively, in outliers.

Non-parametric analysis (Spearman correlation) revealed a number of significant inter-species correlations. Based on log counts, and after performing Bonferroni correction for multiple comparisons (P ≤ 0.002), fusobacteria showed significant correlation with prevotellae and *P. micra* (r = 0.71 and 0.53, respectively), while *T. denticola* significantly correlated with *T. forsythia* (r = 0.72). Almost the same patterns of associations were found based on proportions, but the correlation between fusobacteria and *P. micra* disappeared. Taking a less conservative level of significance (P ≤ 0.01), *T. forsythia* was also found to positively correlate with fusobacteria and prevotellae.

### Periodontitis vs. healthy sites

The absolute log counts of the test species/genera in the subgingival plaque samples from the healthy and periodontitis sites are presented in Table 3. More total bacterial DNA was recovered from the periodontitis sites than from the healthy sites although this did not withstand adjustment for multiple comparisons. All test species/genera, with the exception of *A. actinomycetemcomitans and Fusobacterium* spp*.,* were also present at higher levels at sites with periodontal destruction; however, only *P. micra* and *T. forsythia* maintained significant difference after correction for multiple comparisons (P ≤ 0.0063).

**Table 3:** Median (interquartile range) log counts of the test species/genera in subgingival plaque from the healthy and periodontitis sites

**Species**	**Healthy sites (n = 20)**	**Periodontitis sites (n = 20)**	***P***
Total bacteria	8.65 (8.46-8.83)	8.78 (8.52-9.10)	0.04
*A. actinomycetemcomitans*	2.42 (0.00-3.50)	2.40 (0.00-307)	0.67
*Fusobacterium spp.*	6.62 (5.82-6.85)	6.37 (6.00-7.03)	0.08
*Prevotella spp.*	6.20 (5.65-6.65)	6.42 (6.07-6.80)	0.02
*P. micra*	4.33 (3.78-5.21)	5.40 (4.92-5.65)	0.0001*
*P. gingivalis*	3.90 (2.56-5.70)	5.52 (3.07-6.04)	0.04
*T. forsythia*	6.09 (5.24-6.47)	6.71 (5.97-6.91)	0.006*
*T. denticola*	5.47 (4.10-6.26)	6.16 (5.71-6.76)	0.02

*Statistically significant after adjustment for multiple comparisons *(P* ≤ 0.0063); Wilcoxon signed ranks test.

Using relative counts (proportions), all test species, except *A. actinomycetemcomitans*, were present at higher proportions in the periodontitis sites compared to the healthy sites (Table 4). However, differences were only significant for *P. micra* and *T. denticola* (P ≤ 0.0063) that showed 3.39 and 1.33 median fold increase, respectively, in sites with periodontal destruction compared to sites with no destruction. *P. micra* was present at higher relative counts in periodontitis compared to healthy sites across all subjects.

**Table 4:** Median (interquartile range) relative counts (% total bacteria) of the test species/genera in subgingival plaque from the healthy and periodontitis sites

**Species**	**Healthy sites (n = 20)**	**Periodontitis sites (n = 20)**	**Fold difference**	**P**
*A. actinomycetemcomitans*	0.001 (0.000-0.020)	0.001 (0.000-0.005)	0.07	0.12
*Fusobacterium spp.*	0.789 (0.238-1.205)	0.701 (0.250-1.029)	0.81	0.79
*Prevotella spp.*	0.360 (0.222-0.903)	0.394 (0.228-0.751)	1.23	0.50
*P. micra*	0.009 (0.003-0.022)	0.031 (0.014-0.081)	3.39	0.0001*
*P. gingivalis*	0.004 (0.0001-0.0611)	0.068 (0.0001-0.219)	1.97	0.156
*T. forsythia*	0.302 (0.055-0.541)	0.425 (0.170-1.50)	0.86	0.08
*T. denticola*	0.092 (0.007-0.438)	0.338 (0.126-0.613)	1.33	0.006*

*Statistically significant after adjustment for multiple comparisons (*P* ≤ 0.0063); Wilcoxon signed ranks test.

## Discussion

Studies from the Middle East are limited to those that assessed the effect of traditional oral hygiene such as miswak, or certain habits, like qat chewing, on levels of periodontal pathogens [17,20,21]; no studies have so far assessed which periodontal pathogens are particularly associated with periodontitis in an Arab population. The study compared counts of 7 putative pathogens between healthy and diseased sites in patients with moderate-severe chronic periodontitis, using real-time PCR. Despite the advantage of this techniques (sensitivity and possibility of relative quantification), a limited number of studies used it in the study of microbiology of periodontitis and even fewer used relative quantification for making comparisons between health and disease. Healthy sites within the same subjects rather than healthy individuals were used as controls to avoid the effects of inter-individual variations in factors other than microbial composition. Nevertheless, including healthy subjects as additional controls would have allowed for more comparisons and a broader view of differences in microbial composition between periodontal health and disease. So this could be considered as one limitation of the current study. Another limitation is that bleeding on probing was not recorded although it has been previously shown to be an important clinical variable. In addition, while the most important pathogens were tested, the panel could have included more species particularly the newer pathogens such as *Filifactor alocis*, oral synergistetes and TM7.

Absolute counts were reported in DNA copies rather than cell numbers because the target gene copy number per genome, particularly 16S rRNA gene, varies from one species to another and is not known for some of them. This implies that actual bacterial counts for some of the tested species/genera are less than the counts reported in the study. It, however, does not influence on validity of comparisons between sites or patients. Theoretically, as little as one gene copy per reaction can be detected by real-time PCR; however, in practice this is usually not possible. Some of the previous studies on periodontal pathogens were in fact not clear with respect to whether the reported lower detection limit was per reaction or per sample, but it can be concluded that at least 100 DNA copies per reaction could be detected [11,22]. However, a detection limit of as low as 1.6 cells per reaction has been reported [9,10] which is comparable to the sensitivity of q-PCR assays in this study (5 copies/reaction). The high sensitivity of q-PCR probably explains the higher detection rates of pathogens observed in studies that employed this technique compared to those found with culture techniques or DNA-DNA hybridization.

The median log counts of total bacteria as well as some of the test species/genera in this study are 1–3 log higher than those reported by most previous studies that used real-time PCR [9,10,12,22]. In contrast, Lyons et al. [11] reported counts as high as 10^14^ and 10^12^ for total bacteria and *P. gingivalis*, respectively, which sounds implausible. While these differences can be attributable to variations in sampling and DNA extraction efficiency, inaccuracies in preparation of standard curves probably account for the great part of them. Curves prepared using serial dilutions of bacterial cells or genomic DNA extract, as done in most previous studies, may not be as accurate as those generated using plasmids. On the other hand, quantification bias because of plasmid DNA conformation has been reported recently, especially with circular plasmids [23]. However, since values from this study are also close to those previously obtained by checkerboard DNA-DNA hybridization [24], they can be assumed to be accurate enough.

The relative abundances of *T. denticola* and *T. forsythia* in the current study are very comparable to those reported for a Japanese population using a similar quantification technique [12]. In the latter study, however, *P. gingivalis* and *Prevotella intermedia* were presented at exceedingly higher proportions and showed significant association with the disease which in contrast with the current findings. Using checker board DNA-DNA hybridization, much higher proportions of the red complex members have been reported in chronic periodontitis patients from USA, Brazil, Chili and Sweden [13] compared to the relative counts described here. However, this can be simply justified by the fact that proportions in the checker board technique are calculated by normalizing absolute counts of each species to total counts of the 40 probe species used rather than total bacteria as done in the current study.

Relative quantification data show that although periodontal pathogens were present at significantly higher proportions in periodontitis sites compared to healthy sites, they still constituted a minority of subgingival microbiota. This is, however, not surprising since assessment of earlier cultivation-based studies and more recent studies employing molecular techniques clearly reveals that periodontal pathogens, particularly members of the red complex, have almost always been detected at low abundance [3,24,25]. What has not been addressed adequately, on the other hand, is how these pathogens can cause periodontitis at such low abundance. One interesting, currently evolving view is that low abundance periodontal pathogens orchestrate periodontitis by inducing a dysbiotic “pathogenic” microbial community that in turn mediates bone destruction [26]. This is thought to result from ability of these pathogens to subvert some components of the host response rather than to act directly as proinflammatory bacteria as has been very recently demonstrated for *P. gingivalis* in vitro [27]. Accordingly, low abundant periodontal pathogens are claimed to function as keystone pathogens, a hypothesis that challenges the role of red complex members as conventional pathogens [28].

The current study did not show an association between *P. gingivalis* and periodontal destruction, which is very hard to defend against the existing overwhelming evidence. However, this can simply be a failure to detect existing association due to lack of adequate power, especially that there was a significant difference in absolute count at the 0.05 level. On the other hand, given the polymicrobial nature of periodontitis, and in view of the new keystone pathogen hypothesis, it is also plausible to propose that other members of the pathogenic team can in certain circumstances take over the role of *P. gingivalis* as a keystone pathogen. In fact, *P. gingivalis* has not always showed the strongest association with periodontitis [3,29,30]. In the current study, *T. denticola* and *T. forsythia*, both members of the red complex, did show significant association with periodontitis, which is consistent with the literature. However, *P. micra* (previously known as *Peptostreptococcus micros*) showed the strongest association with the disease being present at significantly higher absolute and relative counts in periodontitis sites in all study subjects. This species is a member of the orange microbial complex [6], and there is an expanding evidence on its role, along with other peptostreptococci, as a periodontal pathogen [3,29,31,32].

## Conclusion

Despite its presence in very low relative counts, *P. micra* showed the strongest association with periodontal destruction, which is suggestive of a potential role as keystone pathogen in place of *P. gingivalis*. However, this needs to be validated in a larger-scale study before it can be claimed to represent ethnic variations in pathogens’ association with periodontitis.

## Competing interests

The authors declare that they have no competing interests.

## Authors’ contributions

NA designed and carried out the laboratory work, performed the statistical analysis of data and wrote the manuscript. HS was responsible of the field work, collection of specimens, and data entry. AA contributed to the study design, supervision of clinical data collection, and writing of the manuscript. IG was involved in the study design, and overall supervision of the research project. All authors read and approved the final manuscript.

## Pre-publication history

The pre-publication history for this paper can be accessed here:

http://www.biomedcentral.com/1472-6831/14/13/prepub
